# 3D Reconstruction of the Digestive System in *Octopus vulgaris* Cuvier, 1797 Embryos and Paralarvae during the First Month of Life

**DOI:** 10.3389/fphys.2017.00462

**Published:** 2017-07-04

**Authors:** Raquel Fernández-Gago, Martin Heß, Heidemarie Gensler, Francisco Rocha

**Affiliations:** ^1^Department of Ecology and Animal Biology, University of VigoVigo, Spain; ^2^Estación de Ciencias Marinas de Toralla (ECIMAT), Marine Science Station of Toralla, University of VigoVigo, Spain; ^3^Biozentrum der Ludwig-Maximilians-Universität München (LMU)Planegg, Germany

**Keywords:** *Octopus vulgaris*, paralarvae, ontogeny, digestive system, 3D

## Abstract

*Octopus vulgaris* aquaculture is limited due to poor biological knowledge of the paralarval stages (e.g., digestive system functionality), their nutritional requirements (e.g., adequate live diet) and standardization of rearing techniques. These factors are important in explaining the high mortality rate observed in this developmental stage under culture conditions. For a better understanding of nutrition biology of this species, we investigated the 3D microanatomy of the digestive tract of the embryo and paralarvae during the first month of life. *O. vulgaris* paralarvae digestive system is similar to that in the adult. The “descending branch” has a dorsal position and is formed by the buccal mass, oesophagus and crop. Ventrally, the “ascending branch” is formed by the intestine and the anus. The digestive gland, the posterior salivary glands and the inner yolk sac (in the case of the embryo and hatched paralarvae) are located between the “ascending” and “descending” branches. In the curve of the U-shaped digestive tract, a caecum and the stomach can be found. The reconstructions reveal that anatomically the digestive system is already complete when the paralarvae hatch. The reconstruction of the buccal mass at different post-hatching days has demonstrated that all the necessary structures for food intake are present. However, the radula surface in contact with the pharynx is very small on the first day of life. Although the digestive system has all the structures to feed, the digestive gland and radula take longer to reach full functionality. We have established four development periods: embryonic, early post-hatching, late post-hatching and juvenile-adult. The differentiation between these periods was done by type of feeding (endogenous or exogenous), the state of maturation and hence functionality of the digestive gland, type of growth (linear, no net, or exponential), and measurement of the arm lengths with respect to the mantle length. 3D reconstruction represents a new tool to study the morphology and functionality of the cephalopod digestive system during the first days of life.

## Introduction

*Octopus vulgaris* aquaculture is limited due to a number of factors including: poor physiological and biological knowledge of the paralarval stages, their nutritional requirements, a live diet with adequate composition, and the standardization of rearing techniques (Moxica et al., 2002; Iglesias et al., 2007; Iglesias and Fuentes, 2014). Despite determined research efforts (Itami et al., 1963; Iglesias et al., 2004; Iglesias and Fuentes, 2014) octopus aquaculture continues to have high mortality during the first month of life. Recent studies have focused on understanding the nutritional requirements in the paralarval stage (Villanueva et al., 2002; Iglesias et al., 2006; Seixas et al., 2008; Reis et al., 2015). However, little research has been undertaken to standardize aquaculture techniques (Moxica et al., 2002; Reis et al., 2015; Iglesias et al., 2004, 2007; Domingues et al., 2010; Fuentes et al., 2011) or to learn about the biology of this developmental stage (Roura, 2013).

Major changes in shape and morphology of the organs occur in the larval stage of development. In teleost ontogeny, these changes in the alimentary tract occur in both morphology and function (Dabrowsky, 1986). This can be observed using methods that detect both, morphological and functional changes. Most larvae have a simple or still undifferentiated digestive tract in the first days of life (Govoni et al., 1986). Changes can include the opening of the mouth, increase in the relative length of the intestine and oesophagus, the formation of the stomach, development of intestinal mucosal foldings and changes in protein digestion or enzymatic activity in general (Porter and Theilacker, 1999; Cahu et al., 2004; Mangetti, 2006). Comparative studies of digestive system development have been performed in a number of fish species including *Solea solea* (Boulhic and Gabaudan, 1992), *Dicentrarchus labrax* (Beccaria et al., 1991), *Dentex dentex* (Crespo et al., 1992), *Pagrus pagrus*, and *Diplodus sargus* (Darias, 2005).

Previous studies of *O. vulgaris* embryonic structures show the organogenesis of the gonad, hepatopancreas and circulatory systems (Naef, 1928; Boletzky, 1967, 1968; Marthy, 1968). Also, general and comparative aspects of the embryonic development have been reported by Boletzky (1969, 1971). Several authors (cited by Boletzky, 1978) have also studied the embryonic digestive system. However, these previous studies focused on the origin and formation of the embryonic structures but do not show the changes occurring between embryonic and paralarval stages. In addition, the digestive system functionality of cephalopods in paralarval stages is poorly known. There are only a few studies concerning the development of the digestive system in cephalopod hatchlings (Moguel et al., 2010; Martinez et al., 2011; López-Peraza et al., 2014). The nutritional requirements in the larval stages are critical in aquaculture (Mangetti, 2006). A comparative study of the embryonic and paralarval developmental stages provides new insights into digestive system morphology that also may facilitate physiological studies of these stages. If the digestive system is underdeveloped or does not possess all the structures capable of digesting the food, its absorption capacity will be limited. This will allow us to better identify the food requirements at the most critical stage of the culture to reduce mortality and to find a specific diet and nutritional protocol for this phase.

The present study describes the anatomical changes in the digestive system of *O. vulgaris* embryos and paralarvae at different phases of its ontogenetic development during the first month of life. This study represents the first 3D reconstruction of octopus paralarvae. In addition, this study describes a new tool to study the functional morphology of cephalopod organs during their first days of life. These data provide an explanation for the adaptation to the use of exogenous food and provide useful information to facilitate aquaculture of this species.

## Materials and methods

### Paralarvae rearing

*Octopus* paralarvae were obtained from the Ría de Vigo on 14th and 22nd August 2013, through the collection of egg strings. These were transported to the ECIMAT (REGA ES360570181401) in tanks with seawater and then placed in 150L tanks with running seawater in the dark until hatching occured. The hatching paralarvae were observed in the laboratory to exclude the selection of premature paralarvae. Subsequent paralarvae rearing was conducted in two cylindrical tanks (150 L) with dark walls. The temperature of the culture was 21°C (Hamasaki and Morioka, 2002), the period of illumination was 12 h, with one water renovation per day and moderate aeration. A total of 1350 paralarvae were reared in each tank. The diet was supplied *ad libitum* each 2 days. This consisted of *Artemia* sp. enriched with *T-iso* and *Rhodomonas lens* over 3 days before they were used. All procedures involving in this study were carried out under Directive 2010/63/EU, in accordance with the recommendations of Bioethics Committee of Santiago de Compostela University (RD. 53/2013 12th of February) and Ethics Committee of Animal Experimentation of Vigo University (Protocol number 10/2013 2nd December).

### Photography and morphometry

We took samples of the eggs in the stages X, XIII, XIX (Naef, 1928) and paralarvae at 5, 7, 9, 12, 15, 20, 29, and 35 post-hatching days. The paralarvae were anesthetized with ethanol prior to photographing to prevent stress suffering during the processing method (Rocha et al., 2015). Each paralarva was extracted individually by siphoning and then it was deposited in a Petri dish with 10 ml of seawater. Subsequently, 96% ethanol drops were added to the seawater to reach a concentration of 1%. No ethanol was added directly on the paralarvae. The signs to consider the individual anesthetized were a cessation of swimming, decreased activity of chromatophores, lack of arm movement, decreased cardiac activity, and relaxation of the musculature of arms and mantle (Gleadall, 2013). Each specimen was maintained anesthetized 5 min to take photos and measures. No toxic effects or indicators of stress were observed during anesthesia, such as ink release, skin and eye irritation, abnormal changes in color and texture of the skin or contraction in body musculature (Andrews et al., 2013; Gleadall, 2013). Image acquisition was performed using a binocular loupe Nikon SMZ 1500. Pre-fixation death was achieved by slowly increasing the ethanol concentration to 10% when the heartbeat ceased. Total length (TL), dorsal mantle length (DML), arm length (AL), and the number of suckers were measured for paralarval stages.

### Semithin section series

**Figure 1:**
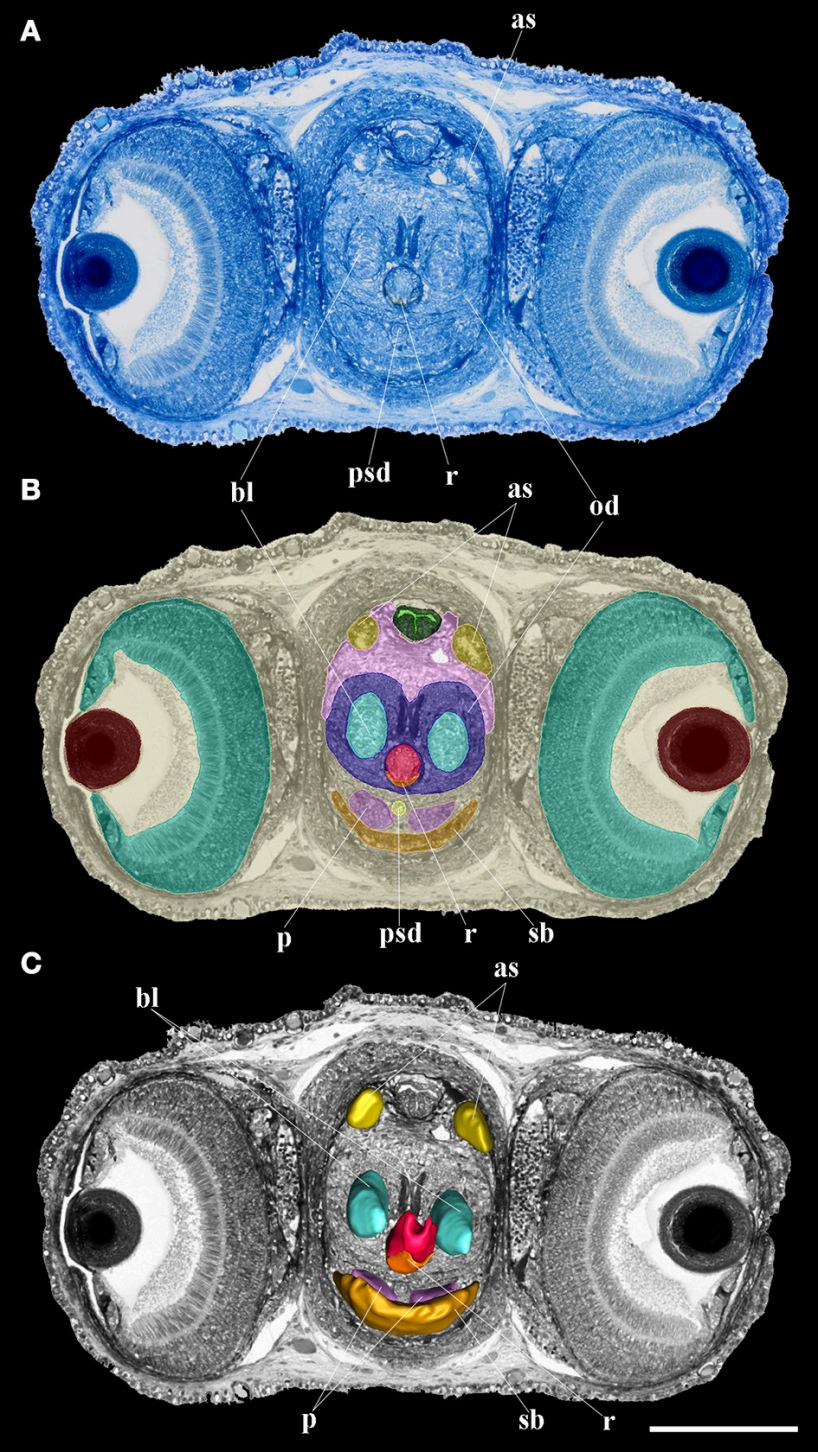
Oral bulb: from histology to reconstruction. **(A)** Semithin section stained in Richardson blue; **(B)** Segmentation of the different oral bulb organs and structures; **(C)** 3D reconstruction of the different oral bulb organs and structures. as, anterior salivary glands; bl, bolster; od, odontophore; p, salivary papilla; psd, posterior salivary duct; r, radula; sb, submandibular gland. Scale bar 200 μm.

For the 3D reconstructions, we selected a single specimen of the embryonic stages X, XIII and for 0, 5, 10, 20, and 35 post-hatching days each. The samples were fixed in 4% glutaraldehyde in 0.1 M cacodylate buffer for several days. Post-fixation was made in osmium tetroxide (1% in cacodylate buffer) and after dehydration, in a graded acetone series, the samples were embedded in epoxy resin following standard protocols. Semithin section series (1.5 μm) were made with an ultramicrotome (RMC MT 7000) with a diamond knife according to the protocol established by Ruthensteiner (2008). The sections were mounted on glass slides and stained with Richardson blue (Figure 1A). Photographs of the complete microscope slides were obtained with an automated Olympus BX61VS light microscope (20x objective) equipped with an Olympus IX2-FCB digital camera and dotSlide system. Using Olympus OlyVIA software (Leadtools, USA) a snapshot of every fourth slice was taken for further digital imaging.

### Computer reconstruction and volumetry

The images were pre-processed in Photoshop 6.0 (San Jose, USA) to change the color to gray-scale and to enhance contrast followed by unsharp masking. The 3D processing was performed with Amira software (FEI, Germany) following the steps described by Ruthensteiner (2008). In brief: for the selection of the different organs and structures, we used the *Segmentation Editor* (Figure 1B). Selections, i.e. tracing the cutting profiles of each histological structure considered, were made manually in every image plane. We used the *SurfaceGen* module to generate smooth 3D surface models (Figure 1C) of these structures by its implemented triangulation algorithm. Images of the 3D models in preferred perspectives and organ reconstructions were obtained using the *Snapshot* tool (see also Laforsch et al., 2012). Volume measurements of organs were made with the *Measurements* module (counting all voxels of each structure and multiply the value by the single voxel volume). We calculated the relative volume of each structure of interest in relation to the “total volume,” i.e., the volume of the animal body and external yolk for embryo and from the dorsal tip of the end of the external lips for paralarvae.

### Statistical analysis

All results are presented as mean ± SD using Microsoft Excel (Microsoft, USA). The total length, the relative and total volume of the digestive system structures curves at different ages were interpolated between the measured values using the graph function in Microsoft Excel (Microsoft, USA). As point trend adjustment measures have been used for the coefficient of correlation, values close to 1 indicate a reliable trend of data.

## Results

**Figure 2:**
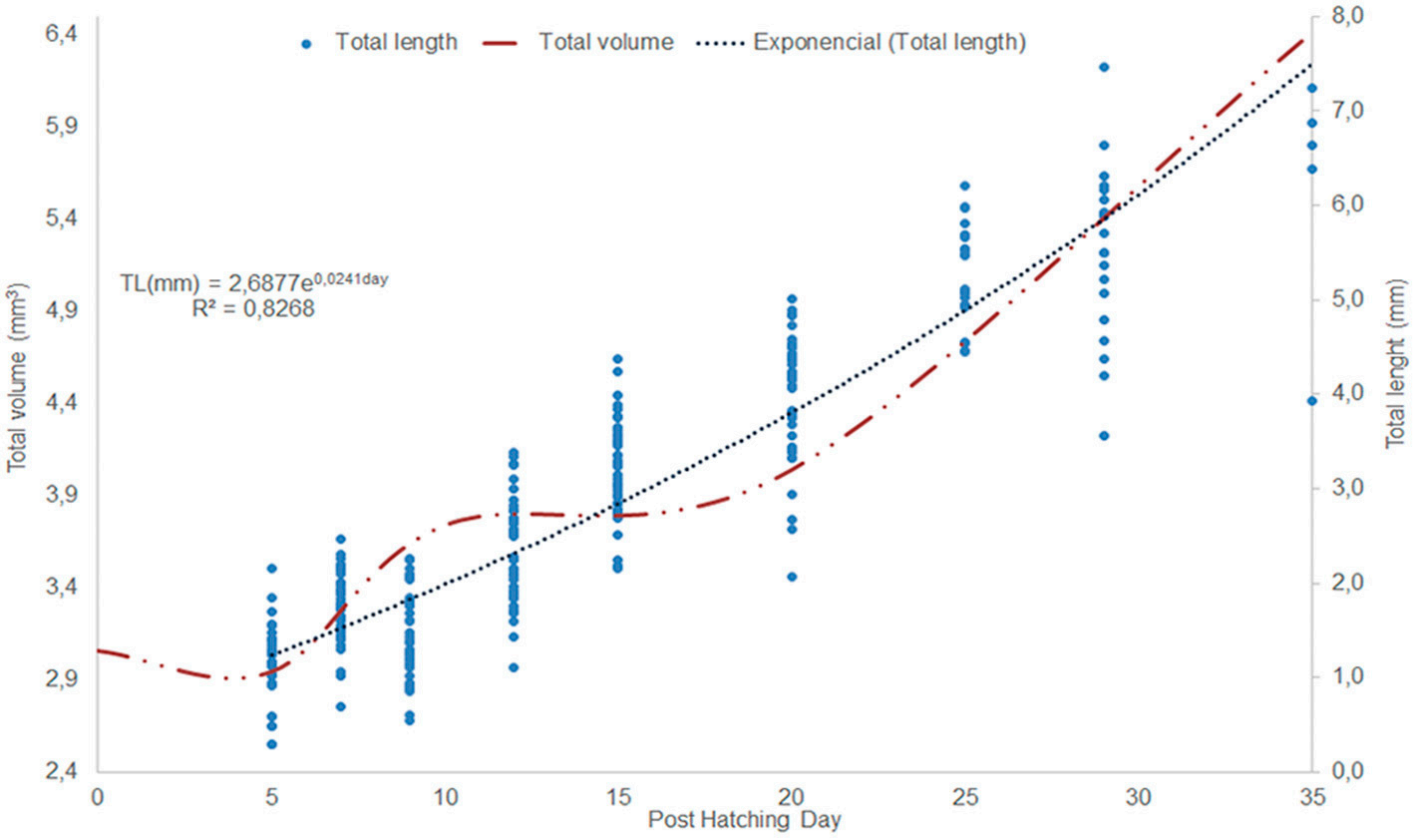
Changes in *Octopus vulgaris* paralarvae total length (mm) and total volume during the first 35 days of life. The dots represent an animal total length measurement.

The morphometric changes of *O. vulgaris* paralarvae show an exponential growth curve from the fifth post-hatching day (Figure 2). The total length (Table 1) at the fifth post-hatching day was 2.99 ± 0.23 mm, the dorsal mantle length was 2.08 ± 0.15 mm and the arms present a mean length of 0.625 ± 0.066 mm with 3 suckers. At the end of the rearing phase (35 days post hatching), the mean total length of the paralarvae was 5.58 ± 0.67 mm, the mean dorsal mantle length was 3.56 ± 0.37 mm and the arm length was 1.44 ± 0.18 mm with 7 functional suckers and 3 primordial suckers.

**Table 1:** The growth of *O. vulgaris* paralarvae from 5–35 post hatching days.

**Stage (age, sample size)**	**Total length (mm)**	**Dorsal mantle length (mm)**	**Mean arm length (mm)**	**Relation DML/AL**	**Suckers**
(5 day, *N* = 30)	2.99 ± 0.23	2.08 ± 0.15	0.625 ± 0.066	3.328	3
(7 day, *N* = 40)	3.29 ± 0.19	2.21 ± 0.14	0.657 ± 0.083	3.364	3
(9 day, *N* = 40)	3.12 ± 0.24	2.22 ± 0.19	0.523 ± 0.098	4.245	3
(12 day, *N* = 40)	3.60 ± 0.29	2.59 ± 0.19	0.706 ± 0.101	3.669	4
(15 day, *N* = 40)	4.03 ± 0.21	2.78 ± 0.21	0.789 ± 0.104	3.523	5
(20 day, *N* = 40)	4.43 ± 0.22	3.04 ± 0.22	0.912 ± 0.135	3.333	5
(25 day, *N* = 20)	5.10 ± 0.28	3.41 ± 0.22	1.129 ± 0.163	3.020	6
(29 day, *N* = 20)	5.22 ± 0.47	3.40 ± 0.25	1.278 ± 0.213	2.660	7
(35 day, *N* = 5)	5.58 ± 0.67	3.56 ± 0.37	1.44 ± 0.180	2.472	7

**Figure 3:**
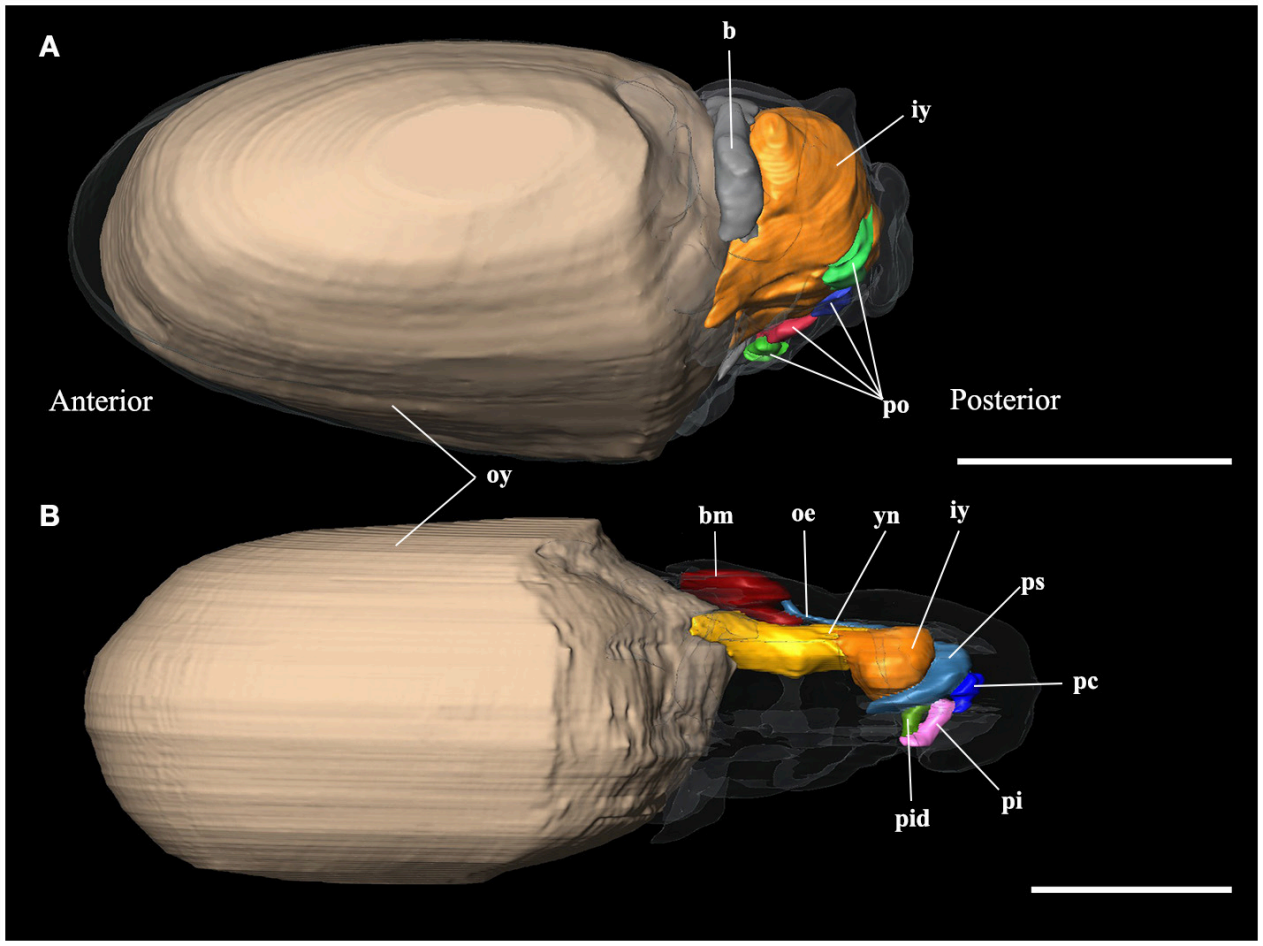
3D reconstructions of the digestive system of embryo stages of *Octopus vulgaris*. **(A)** Stage X of Naef (1928); **(B)** Stage XIII of Naef (1928); b, brain; bm, primordium of the buccal mass; iy, inner yolk; oe, oesophagus; oy, outer yolk; pc, primordium of the caecum; pi, primordium of the intestine; pid, ink duct and ink sac primordium; po, promordia of different organs; ps; posterior salivary glands; yn, yolk neck. Scale bars 500 μm.

**Figure 4:**
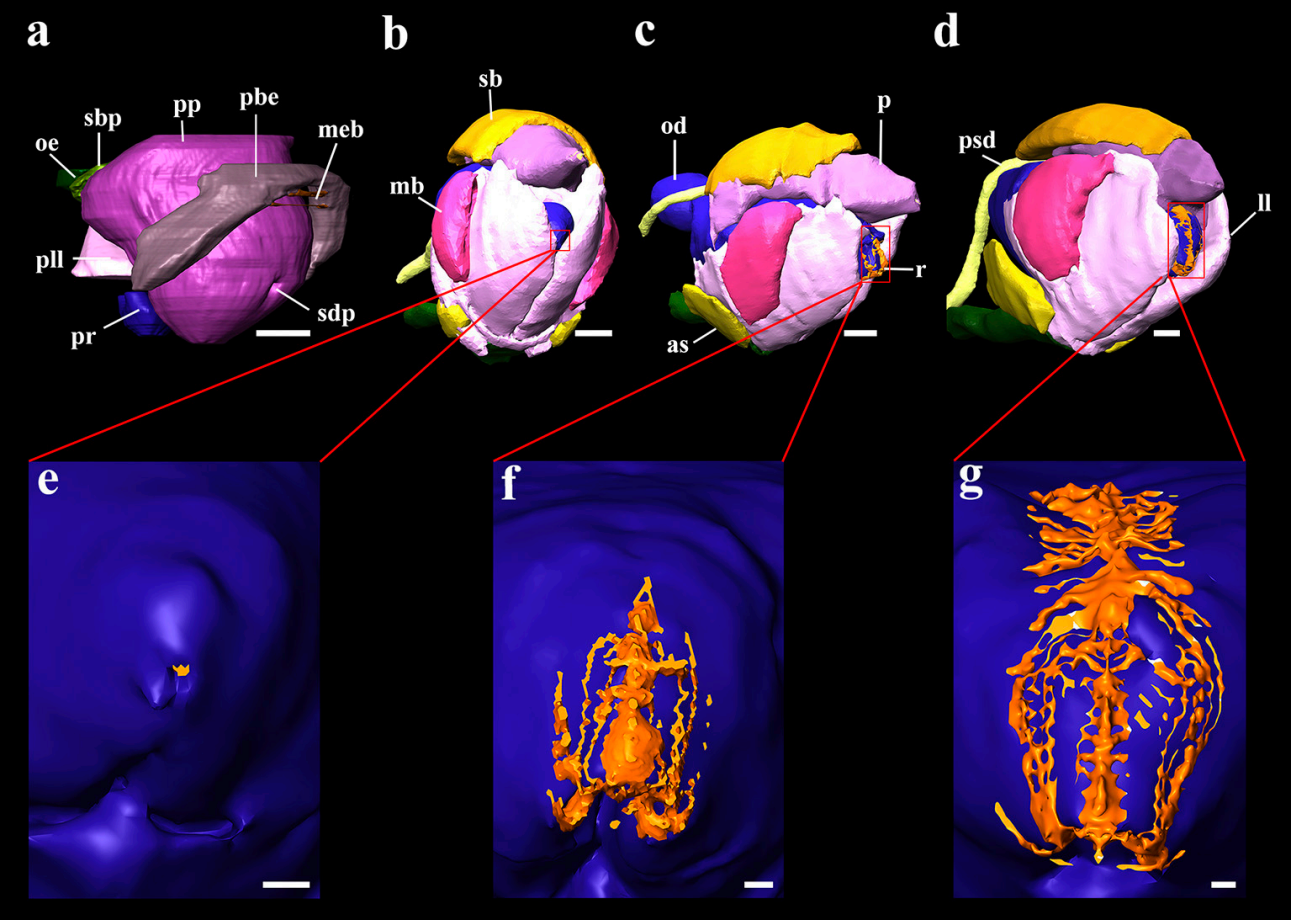
3D reconstructions of the buccal mass and odontophores of *Octopus vulgaris* embryos and paralarvae at different development stages. **(a)** Primordium of buccal mass at stage XIII of Naef (1928); **(b)** buccal mass of the hatching paralarva; **(c)** buccal mass of 5 days post hatching paralarva; **(d)** buccal mass of 20 days post hatching paralarva; **(e)** odontophore and surface of the radula in contact to pharynx in the hatching paralarva; **(f)** odontophore and surface of the radula in contact to pharynx in 5 days post hatching paralarva; **(g)** odontophore and surface of the radula in contact to pharynx in 20 days post hatching paralarva. as, anterior salivary glands; ll, lateral lobes; mb, muscles of beaks; meb, membrane of beaks; od, odontophore; oe, oesophagus; p, salivary papilla; pbe, primordium of beaks; pll, primordium of lateral lobes; pp, salivary papilla primordium; pr, odontophore primordium; psd, posterior salivary duct; r, radula; sb, submandibular gland; sbp, submandibular gland primordium; sdp, salivary duct primordium. Scale bars (**a–d**) 50 μm; Scale bar (e-g) 10 μm.

**Figure 5:**
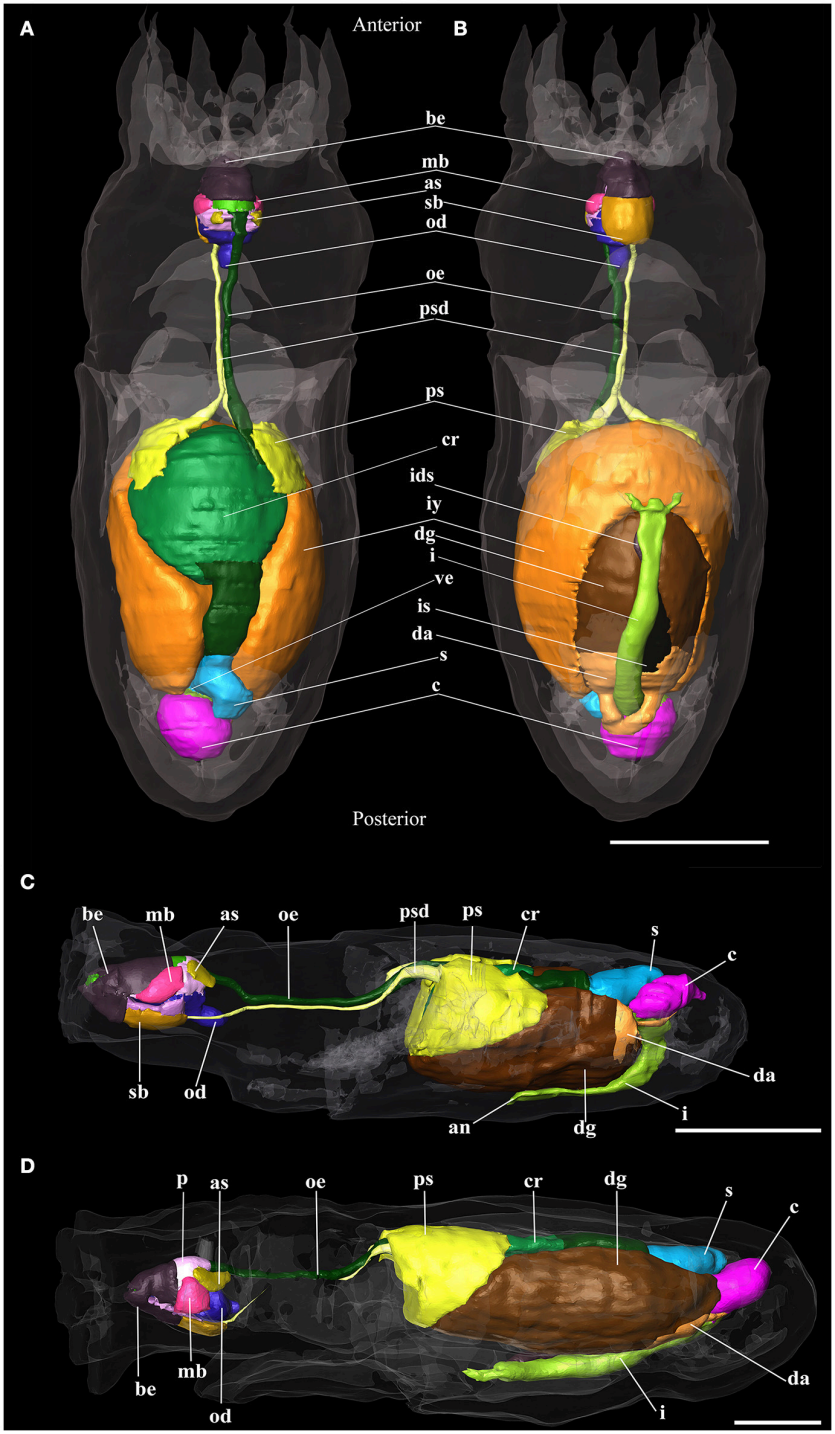
3D reconstructions of paralarva digestive system of *Octopus vulgaris*. **(A)** Dorsal view and **(B)** Ventral view of hatching paralarvae; **(C)** 5 days old paralarva reconstruction; **(D)** 30 days old paralarva reconstruction. an, anus; as, anterior salivary glands; be, beaks; c, caecum; cr, crop; da, digestive appendages; dg, digestive gland; i, intestine; ids, ink sac duct; is, ink sac; iy, internal yolk; mb, muscles of beaks; od, odontophore; oe, oesophagus; p, salivary papilla; ps, posterior salivary glands; psd, posterior salivary gland duct; s, stomach; sb, submandibular gland; ve, vestibule. Scale bars **(A,B)** 100 μm; Scale bars **(C,D)** 500 μm.

The **embryo** reconstructions (Figures 3A,B) show different yolk absorption periods. Stage X of development (Figure 3A) shows outer and inner yolk: while the outer yolk represents 89% of the total animal volume, the inner yolk contributes only 5%. The connection between these two structures is wide, the only difference in these structures is their position inside or outside the embryo. The inner yolk is not bilobulated. The reconstructions of this stage show the second absorption period according to Portmann and Bidder (1929). In this stage, it is possible to see the brain primordium which is surrounded by the anterior part of inner yolk. The XIII Naef's stage reconstruction (Figure 3A) represents the third yolk absorption period. The yolk is represented by inner (1% of total body volume) and outer yolk (82%). The connection between them is made by a narrow neck and the inner yolk has a bilobulated shape. This structure is not in connection with any part of digestive primordium. In the anterior region, it is limited by the brain, eyes and statocysts. At the posterior region, it is limited by the still undifferentiated digestive tract. At this stage, the onset of oral bulb differentiation is observed (Figures 3B, 4a). This consists of the following structures: the outer lips, the primordia of the beak, salivary papilla, and odontophore. The reconstruction of embryo stage XIX (not shown) represents the last stage of the third period of yolk absorption: In this stage, the yolk is only represented by the inner yolk. The digestive system is similar to day 0 (i.e., just hatched) paralarvae (Figures 5A,B).

The digestive system of *O. vulgaris* **paralarvae at hatching** already has a structure like that of the adult (Figures 5A,B). The “descending branch” has a dorsal position and it goes from the anterior to the posterior region of the paralarva. It is formed by the buccal mass, oesophagus, and crop. The “ascending branch,” which is situated ventrally and runs from the posterior to the anterior region of the paralarva, is formed by the intestine and the anus. Between both branches, the digestive gland, the posterior salivary glands and the inner yolk are located. The latter structure is not connected with the digestive gland. In the curve of the U, the caecum and the stomach can be found.

The oral bulb or buccal mass of this stage already has all the structures necessary for food intake (Figures 4b,e), although some of them are not yet fully developed. The mouth is completely open to the outside, showing no membrane that would block the entry of food. The center is occupied by the salivary papilla and the radula. All these structures are surrounded by the beak. The radula surface in contact with the pharynx is very small on the first day of life (see **Table 3**). The salivary glands, anterior and posterior, are connected to the buccal mass. The latter structure is connected with the stomach by the oesophagus. It is a narrow tube which, at the level of the posterior salivary glands, gets wider posteriorly and finally opens into the crop (Figure 5A). The stomach, caecum and the intestine are connected through the vestibule. The intestine is a narrow uncoiled tube that starts in the vestibule, and at the end of it, two papillae and the opening of the ink duct are present (Figure 5B). This structure begins in the dorsal region of the animal and ends in the ventral region after turning back on itself. From the first day, all the major glands are present. The digestive gland is the largest gland of the digestive system with a volume of 4.4% of total body volume. This gland has two defined regions: the glandular region and the digestive appendages in the posterior region. It connects to the caecum by two ducts which unify before entering it (Figure 5B). The posterior salivary glands are the next smallest glands with a volume fraction of 0.67%, located dorsally in the digestive gland. These glands are connected to the oral bulb through the salivary ducts. These ducts merge and have a common opening near the entrance of the mouth (Figure 4B). The other two glands present in the digestive system are the anterior salivary glands and submandibular gland (Figures 4b, 5A), while the first pair is externally to the buccal mass the submandibular gland is embedded in the buccal mass.

**Paralarvae at 5 and 35 post-hatching days** have an even more elongated body shape (Figures 5C,D) and display an increasing complexity of inner organs (Table 2). However, the internal yolk is no longer present at the 5 post-hatching days. Oral bulb reconstruction at different ontogenetic phases (Figure 4) demonstrates that at hatching day the radula (Figures 4b,e) is present but represented by a small undeveloped structure. The volume, contact surface with pharynx and the proliferation of teeth increase with age. This has a considerable growth increment from the first day of life until the 5th day (Table 2). The 3D reconstruction allows us to see the differentiation of this structure with age (Figures 4c,d,f,g). The intestine remains uncoiled in the examined developmental stages at least until posthatching day 35. At day 35 the radula structure is similar to that of the adult.

**Table 2:** Absolute volume (mm^3^) of the digestive system structures and organs and total body volume from the end of the mantle until the end of the external lips.

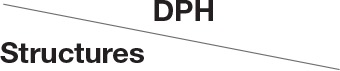	**0**	**5**	**10**	**20**	**35**
Anterior salivary glands	0.0001	0.0006	0.0010	0.0017	0.0043
Posterior salivary glands	0.0089	0.0255	0.0532	0.0645	0.1333
Submandibular gland	0.0007	0.0013	0.0020	0.0036	0.0069
Digestive gland	0.0567	0.1140	0.2846	0.4390	0.7469
Oesophagus	0.0037	0.0030	0.0090	0.0158	0.0285
Stomach	0.0033	0.0051	0.0088	0.0141	0.0400
Caecum	0.0032	0.0034	0.0107	0.0100	0.0279
Crop	0.0160	0.0039	0.0076	0.0103	0.0504
Intestine	0.0033	0.0038	0.0107	0.0160	0.0436
Odontophore	0.0045	0.0068	0.0131	0.0156	0.0327
Radula	0.0001	0.0001	0.0002	0.0002	Broke
Total volume	1.2805	1.0574	2.6110	3.1997	7.8395

*DPH, days post hatching*.

**Figure 6:**
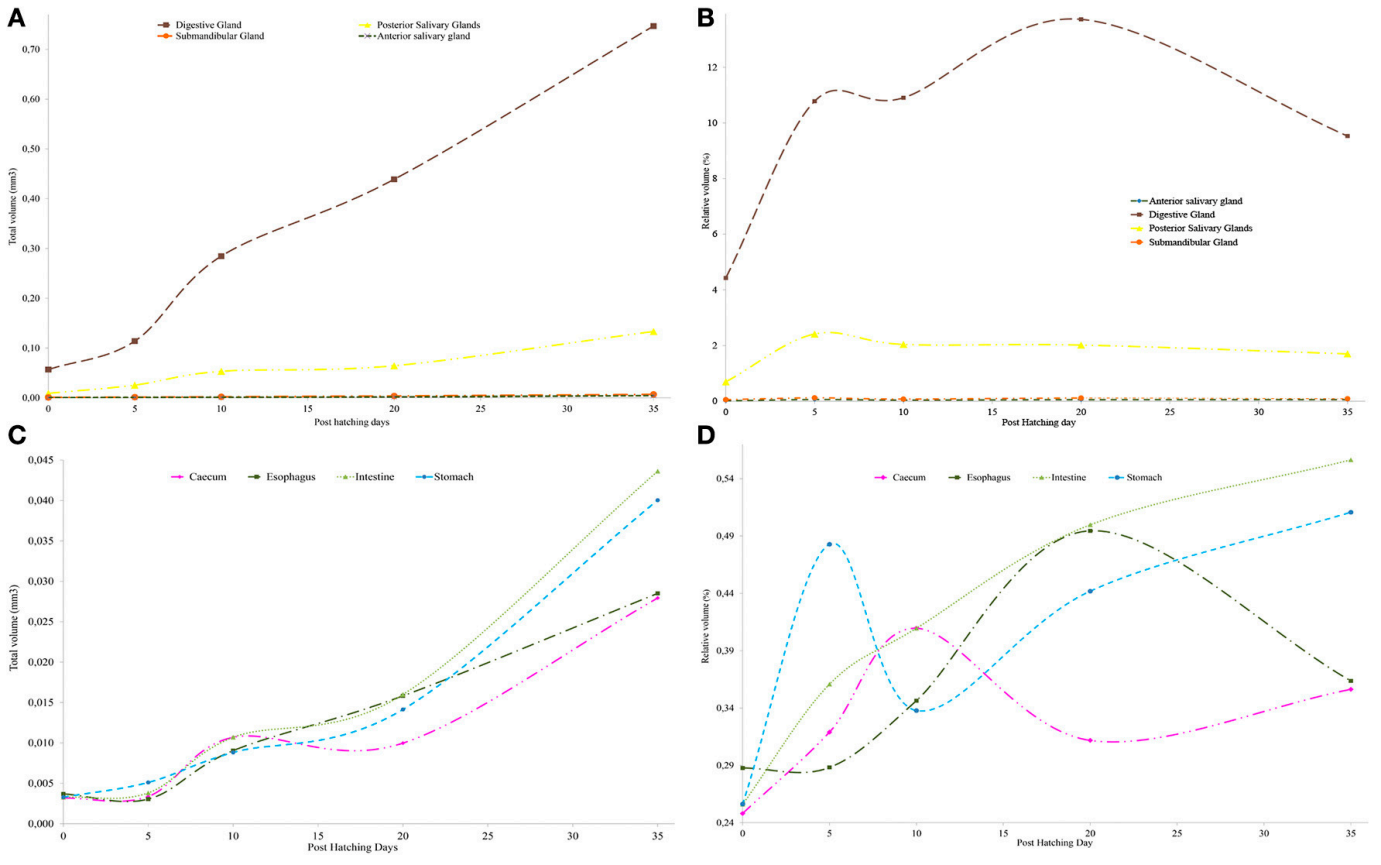
Changes in relative (%) and total volume (mm^3^) of the different digestive system organs. **(A)** Changes in glands absolute volume; **(B)** changes in glands relative volume **(C)** changes in the absolute volume of organs. **(D)** Changes in the relative volume of organs.

The creation of 3D models of the digestive system at different stages of paralarvae development (Figure 4) allowed us to measure total volumes (Table 2) and to observe significant differences in the relative volumes of the internal structures (Table 3). All considered structures have a positive allometry (Figure 6). The structures that have a higher growth in the first month of life are the digestive gland, posterior salivary glands (Figure 6A) and the radula surface, which is in contact with the pharynx (Figures 4e–g). The major increment in the volume of the glands occurs from 10 to 35 post-hatching days. The relative volumes of these structures show the same trend (Figure 6B). However, the total volume of the organs (Figure 6C) show two different growth periods. The greater increments occur from 5 to 10 days and from 20 to 35 post hatchings days. The relative volumes of these structures show that the development of these structures does not have the same trend. In the case of the oesophagus and intestine, the volumes increase from the first day of life until the end of the rearing period (35 days) in the present study. However, the caecum and stomach volume do not show t a clear trend (Figure 6D). The paralarvae total volume (Figure 2) shows a no net growth phase (Vidal et al., 2002) from 0 to 5 post-hatching days. After this time, the total volume of paralarvae shows two different growth periods. The first from 5 to 12 post-hatching days, and the second from day 15 to the end of the rearing.

**Table 3:** Relative volume (%) of the digestive system structures and organs.

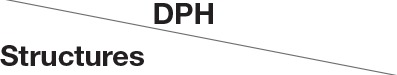	**0**	**5**	**10**	**20**	**35**
Anterior salivary glands	0.012	0.059	0.039	0.054	0.056
Posterior salivary glands	0.695	2.413	2.038	2.015	1.700
Submandibular gland	0.055	0.120	0.075	0.112	0.087
Digestive gland	4.427	10.778	10.920	13.720	9.527
Oesophagus	0.288	0.288	0.346	0.495	0.364
Stomach	0.256	0.483	0.338	0.442	0.511
Caecum	0.248	0.319	0.410	0.312	0.356
Crop	1.248	0.369	0.290	0.323	0.642
Intestine	0.256	0.361	0.410	0.500	0.556
Odontophore	0.355	0.640	0.504	0.487	0.418
Radula	0.008	0.014	0.008	0.007	Broke
Radula in contact with pharynx	0.728	34.651	42.86	35.804	Broke

*DPH, days post hatching. The relative volume is calculated considering the total volume from the end of the mantle until the end of the external lips. The “radula in contact with the pharynx” relative to the absolute radula volume*.

## Discussion

*Octopus vulgaris* completes embryonic development as a planktonic stage called paralarva (Young and Harman, 1988). During this post-hatching stage, the transition between endogenous and the exogenous feeding occurs, which is considered as a critical period characterized by high mortality (Iglesias and Fuentes, 2014). This study provides the first visualization of the anatomical ontogeny of the digestive system from embryonic stage X to the 35 days post hatching paralarvae. This was performed using 3D reconstruction of the structures that make up the digestive system at different stages.

Boucaud-Camou and Boucher-Rodoni (1983) established three developmental phases in cephalopods called: embryonic (1), post-hatching (2) and juvenile-adult (3). The differentiation between these 3 phases lies in the type of feeding (endogenous or exogenous) and the state of maturation and enzymatic activity of the digestive gland. Moguel et al. (2010) have added more features to differentiate these phases including the growth pattern, the length of the arms relative to the length of the mantle and the response to prey. The embryonic stage (1) is characterized by an intracellular digestion of the yolk, linear growth, and weak activity of digestive gland. The post-hatching phase (2) starts with exogenous feeding, in which the size of the digestive gland increases and its enzymatic activity is initiated (Villanueva et al., 2002). Despite the beginning of enzymatic activity, this gland is still relatively immature and erratic function of enzymes occurs (Villanueva et al., 2002; Moguel et al., 2010), with lapses of enzyme synthesis in the digestive gland cells. The inner yolk is still present; this implies both exogenous and endogenous feeding occur at the same time. This phase has a no net growth (Vidal et al., 2002) and the arms are shorter than the mantle thus limiting the ability to capture prey. In the last phase, juvenile-adult (3), the digestive gland appears fully developed with stable enzyme activity (Villanueva et al., 2002). In this phase, the growth is exponential and arms get longer than the mantle. These phases have been identified for cephalopods species with direct development, in which the embryo hatches with characteristics very similar to the adults, as *O. maya* (Moguel et al., 2010).

### Anatomical and morphological changes

The embryonic development stages described here coincide with those described previously by Naef (1928) for *O. vulgaris*, based on the visible anatomical development of structures through the chorion. However, the development of the 3D technique gives much more information about the development of this species. These types of 3D model allow simultaneous analysis of both external structures and internal structures. This allows inferences to be made about development and physiology.

Our results confirm that in *O. vulgaris* the embryonic phase finishes when the animal hatches for nutritive reasons (represented here by the reconstruction of post-hatching day 0 paralarvae). At day 0 paralarvae have the capacity to start exogenous feeding although the paralarvae carry out very few attacks maybe due to the fact that they are not physiologically ready to start their exogenous feeding (Iglesias et al., 2006). Yet, in the first days of life, the paralarvae no longer depend on the yolk resources only, rather they progressively change to a mixed feeding depending on both, the yolk and exogenous sources. In this phase, we can see the inner yolk, an open mouth, small radula (radula surface in contact with the pharynx only 0.73% of the total radula surface), and small digestive gland. Other important structures for prey capture are the arms. At hatching day, they are shorter than the mantle and have only 3 suckers. Although food is available, the animal may have a limited ability to capture it because the arms are relatively undeveloped. Overall, these features could reduce the capacity of the paralarvae to capture and ingest prey.

The duration of this phase is directly related to the incubation and rearing temperature of the paralarvae. Vidal et al. (2005) showed in squid that higher temperatures increase metabolic rates. In our case (21°C) this early post-hatching phase extends from the first day of life to the fifth rearing day. At that day, the inner yolk is depleted, hence its volume is zero, and the paralarvae can only exploit the resource of the exogenous food. Besides, the combination of total length and the total volume of the paralarvae allows us to differentiate the no net growth phase. For all these characteristics, we refer to the “early post-hatching phase” as the period from hatching to 5 days post hatching at 21°C.

We conclude that a late post-hatching phase occurs. In this phase, paralarvae have characteristics intermediate between the early post-hatching and juvenile-adult phases. This is characterized by exogenous feeding, an exponential growth, arms still shorter than mantle length, and increase in the digestive gland size. In this phase, the organism becomes better prepared for effective autonomous feeding (catching success, prey size). The capacity of paralarvae to feed exclusively on exogenous food depends on the ability to find and capture prey (Moxica et al., 2002). The reconstruction of the buccal mass shows that all the necessary structures to actively feed are present. At the fifth day post hatching the radula surface, in contact with the pharynx, increased considerably (34.62%). This enables the animal to more efficiently consume prey. The activity of proteolytic enzymes, however, may be still somewhat limited (see below). In order to define the end of this phase and the starting point of the juvenile phase, it will be necessary to carry out a more detailed histological and histochemical study of the digestive gland to establish when it is completely developed. However, better exogenous food adaptation is demonstrated by the positive allometry of the different structures and total length.

In conclusion, the present study shows that the first 20 days of life in *O. vulgaris* are a transition period until juvenile life starts. In this time of transition, the animal will adapt to the new environmental conditions in which it will live, and we propose four developmental phases in *O. vulgaris*: embryonic, early post-hatching, late post-hatching and juvenile-adult phase. A critical period is represented by the transition between the early and late post-hatching phase.

### Potential physiological implications

The morphological findings described and discussed above, e.g., changes in the absolute size and relative volumes of all parts of the digestive system of *O. vulgaris* paralarvae in combination with the changing possibilities to use endogenous and/or exogenous food forebode changes in, or maturation of, physiological processes during development. These physiological processes, of course, have to be proven in future physiological experiments.

#### Early post-hatching phase

Although the internal yolk is in contact with the digestive gland in just hatched *O. vulgaris* paralarvae, no specialized structure connects these two organs. Portmann and Bidder (1929) established that in the hatching *Loligo* sp. paralarvae, called period III, the yolk nutrients passage is directly to the digestive gland, however, Boletzky (1975) stated that the passage of nutrients from the yolk syncytium directly into the digestive gland is inconceivable and the nutrient absorption is through the posterior sinus. The hatched paralarvae 3D reconstruction presented here cannot confirm Boletzky (1975) or Portmann and Bidder (1929) theory about the yolk absorption. Anyway, in a future study, it has to be clarified when exactly (between D0 and D5) the internal yolk is depleted and how the anatomical relation to the posterior sinus changes.

#### Late post-hatching phase

In this phase, the fast growth of the digestive gland points to the still immature activity of this gland (Villanueva et al., 2002). The transition between this phase and the juvenile phase should be investigated to look for a mature tubule structure and the presence of boules in the cells (Bidder, 1966). These digestive gland structures could indicate maturation of digestive gland function. In other cephalopods paralarvae as *O. maya* (Lopez-Ripoll, 2010) and *S. officinalis* (Boucaud-Camou and Yim, 1980) is observed that the 30D post hatching digestive gland has a more complex tubular structure than 5D post hatching paralarvae.

The juvenile phase starts when the digestive system is matured morphologically and physiologically. Our study demonstrates that after 20 rearing days the rate of increase in the size of this gland slows down. This is in accordance with previous studies on the digestive enzymes in *O. vulgaris* paralarvae where Villanueva et al. (2002) found that at 20 rearing days total proteolytic activity was stabilized, suggesting that by 20 rearing days at 21°C the digestive gland is fully developed and functional.

Considering all that has been said about the development of the digestive system and the use of the yolk in *O. vulgaris*, the early post-hatching phase must be considered critical. This, because it represents the transition from endogenous to exogenous feeding, at a time when some structures of the digestive system are not fully developed. The cephalopod paralarvae are active predators from the time of hatching (Iglesias et al., 2006). Thus, choice of the correct prey as food during the first days of cultivation is critical. In this sense, probably an initial diet based on larvae of decapod crustaceans (*zoeas*) might be more adequate than one based on *Artemia* (Iglesias et al., 2007; Iglesias and Fuentes, 2014).

## Author contributions

RF: Conception and design of study. RF and HG: Acquisition of data. RF and MH: Analysis and/or interpretation of data. RF and MH: Drafting the manuscript. RF, MH, and FR: revising the manuscript critically for important intellectual content. RF, MH, HG, and FR: Approval of the version of the manuscript to be published.

### Conflict of interest statement

The authors declare that the research was conducted in the absence of any commercial or financial relationships that could be construed as a potential conflict of interest.
